# Conversion Chemotherapy With a Modified FLOX Regimen for Borderline or Unresectable Liver Metastases From Colorectal Cancer: An Alternative for Limited-Resources Settings

**DOI:** 10.1200/JGO.19.00180

**Published:** 2019-09-03

**Authors:** Renata Colombo Bonadio, Paulo Henrique Amor Divino, Jorge Santiago Madero Obando, Karolina Cayres Alvino Lima, Débora Zachello Recchimuzzi, Jaime Arthur Pirola Kruger, Daniel Fernandes Saragiotto, Fernanda C. Capareli, Paulo M. Hoff

**Affiliations:** ^1^ Instituto do Cancer do Estado de São Paulo, São Paulo, Brazil; ^2^ Oncologia D’or, São Paulo, Brazil; ^3^ Hospital Sírio Libanês, São Paulo, Brazil

## Abstract

**PURPOSE:**

Conversion chemotherapy is often used for borderline or unresectable (B/U) liver metastases from colorectal cancer (CRC) with the aim of achieving resectability. Although intensive and costly regimens are often used, the best regimen in this scenario remains unclear. We aimed to evaluate the outcomes of patients with B/U liver metastases from CRC treated with conversion chemotherapy with the modified fluorouracil, leucovorin, and oxaliplatin (mFLOX) regimen followed by metastasectomy.

**METHODS:**

We performed a single-center retrospective analysis of patients with B/U liver metastases from CRC treated with chemotherapy with the mFLOX regimen followed by surgery. B/U disease was defined as at least one of the following: more than four lesions, involvement of hepatic artery or portal vein, or involvement of biliary structure.

**RESULTS:**

Fifty-four consecutive patients who met our criteria for B/U liver metastases were evaluated. Thirty-five patients (64%) had more than four liver lesions, 16 (29%) had key vascular structure involvement, and 16 (29%) had biliary involvement. After chemotherapy, all patients had surgery and 42 (77%) had R0 resection. After a median follow-up of 37.2 months, median progression-free survival (PFS) was 16.9 months and median overall survival (OS) was 68.3 months. R1-R2 resections were associated with worse PFS and OS compared with R0 resection (PFS: hazard ratio, 2.65; *P* = .007; OS: hazard ratio, 2.90; *P* = .014).

**CONCLUSION:**

Treatment of B/U liver metastases from CRC with conversion chemotherapy using mFLOX regimen followed by surgical resection was associated with a high R0 resection rate and favorable survival outcomes. On the basis of our results, we consider mFLOX a low-cost option for conversion chemotherapy among other options that have been proposed.

## INTRODUCTION

Colorectal cancer (CRC) remains the second leading cause of cancer death worldwide despite improvements in treatment over the last few years.^1^ The liver is the most frequent site of CRC metastases and is affected in almost 60% of patients with metastatic disease. However, selected patients amenable to complete resection can undergo surgery, which offers improved survival and sometimes cure.^2-4^

Among patients with liver metastases, borderline or unresectable (B/U) metastases are common, even in the absence of metastases in other sites. In this situation, conversion chemotherapy plays an essential role and is used with the intention of reducing liver lesions and allowing resection.^2^ However, data are still scarce on the optimal selection criteria for conversion chemotherapy and which chemotherapy regimen is best in this scenario.

Chemotherapy regimens containing a fluoropyrimidine in combination with oxaliplatin or irinotecan are standard first-line regimens for metastatic disease.^3-5^ Considering the efficacy of these drugs in CRC, they are often included in conversion chemotherapy regimens. Intensive regimens are often used that aim to achieve higher response rates and a greater reduction of liver metastases. For example, one alternative is a combination of infusional fluorouracil, leucovorin, and oxaliplatin (FOLFOX).^3^ The addition of a third cytotoxic agent is associated with even higher response rates.^6-10^ The third cytotoxic agent added to FOLFOX might be the chemotherapeutic drug irinotecan (as in FOLFOXIRI),^11^ a vascular endothelial growth factor (VEGF) monoclonal antibody,^6,12,^ or an epidermal growth factor receptor (EGFR) monoclonal antibody for patients with wild-type *KRAS*.^7,8^

However, the real impact of this intensification of conversion chemotherapy on the surgical outcomes is not clear. In the context of resectable liver metastases, results of phase III trials showed that perioperative chemotherapy with FOLFOX was associated with a benefit in progression free-survival (PFS),^9^ but the addition of cetuximab to chemotherapy had a detrimental effect on PFS.^10^ A review of the studies on B/U liver metastases suggested that the response rate has a strong correlation with the resection rate (*r* = 0.96; *P* = .002).^13^

Another concern is that possible treatment toxicities could compromise liver resection. Oxaliplatin has been associated with vascular changes and sinusoidal obstruction syndrome,^14^ whereas irinotecan can cause steatohepatitis that can increase the 90-day mortality rate after surgery.^15^ When using anti-VEGF monoclonal antibodies, wound healing complications are increased, and elective surgery should be avoided within 28 days of the last dose of bevacizumab.^16^

When the pharmacy budget is low, as it is in limited-resources settings, effective and accessible regimens should be offered as a reasonable and economically viable alternative. The modified regimen is a combination of bolus fluorouracil, leucovorin, and oxaliplatin (mFLOX). This regimen does not require the use of an infusion pump and has a lower cost than the other regimens mentioned earlier.^17^ We aimed to evaluate surgical and survival outcomes of patients with B/U liver metastases from CRC treated by using conversion chemotherapy with mFLOX followed by surgical resection.

## METHODS

### Study Design and Participants

We searched our prospective liver surgery data bank, and study data were collected using the Research Electronic Data Capture—REDCap—tools hosted at Instituto do Cancer do Estado de São Paulo.^18^ We performed a retrospective analysis of consecutive patients with B/U liver metastases from CRC who received chemotherapy with mFLOX followed by hepatic metastasectomy between June 2009 and July 2017 in a single academic cancer center. Patients were excluded if they presented with evidence of metastatic disease in sites other than the liver.

On the basis of data from the literature,^11^ we defined liver metastases as B/U if at least one of the following was present: more than four liver metastases, involvement of the hepatic artery or portal vein, or involvement of the biliary duct. A trained radiologist reviewed radiologic images (computed tomography or magnetic resonance imaging scans of the abdomen) taken before the conversion chemotherapy to assess the resectability criteria.

Electronic medical records were reviewed to collect data on patients’ clinical characteristics, surgical outcomes (results, complications, and margin clearance), and oncologic long-term outcomes (overall survival [OS] and PFS). All patients who died have the date of death registered in the electronic medical records. The study was approved by the local ethics committees.

### Treatment

Patients received conversion chemotherapy with mFLOX, which consisted of a once-per-week bolus of fluorouracil (500 mg/m^2^) and leucovorin (20 mg/m^2^) for 6 consecutive weeks and oxaliplatin (85 mg/m^2^) at weeks 1, 3, and 5 once every 8 weeks. The number of chemotherapy cycles varied according to physicians’ discretion and was based on obtaining sufficient response to allow resection. After chemotherapy, all patients included in this study underwent surgery for resection of the liver metastases. Other complementary treatment strategies such as portal vein embolization and radiofrequency ablation were allowed if they were indicated.

### Statistical Analysis

The primary end point of this retrospective study was OS after surgery. Secondary end points were R0 resection rate, pathologic complete response rate, PFS, prognostic factors associated with PFS and OS, and complications potentially related to chemotherapy after surgery. Descriptive statistics were used to present clinical characteristics and surgical results. Categorical variables are presented as absolute numbers and percentages. Continuous variables are presented using median and range. R0 resection rate consists of the rate of patients with no residual disease after surgical resection, R1 represents microscopic residual disease, and R2 represents macroscopic residual disease.

The Kaplan-Meier method was used for survival analysis. OS was defined as the time from surgery until death as a result of any cause, and PFS was defined as the time from surgery until disease progression or death. Patients who did not experience these events were censored at the time of last follow-up. Prognostic factors associated with OS and PFS were evaluated by using Cox proportional hazards regression. The prognostic factors evaluated were number of liver metastases (> 4 *v* ≤ 4), vascular involvement (yes *v* no), biliary involvement (yes *v* no), result of surgical resection (R1-R2 *v* R0), number of conversion chemotherapy cycles, and conversion chemotherapy dose reduction (yes *v* no). *P* values < .05 were considered statistically significant. All statistical analyses were performed with STATA software version 14 (STATA, College Station, TX).

## RESULTS

### Patients’ Characteristics and Treatment Received

A total of 54 consecutive patients were included in the study. Median age was 60.4 years (range, 23.5 to 77.2 years) and the majority (n = 35; 64.8%) presented with more than four liver metastases (range, 1 to 25 metastases). Sixteen patients (29.6%) had hepatic artery or portal vein involvement, and 16 (29.6%) had biliary involvement. Patients’ characteristics are summarized in Table 1. The median number of chemotherapy cycles before surgery was two (range, one to seven). Eight patients (14.8%) required a dose reduction of conversion chemotherapy. Four patients underwent staged hepatectomies to achieve complete resection. Regarding complementary treatment strategies, nine patients received portal vein embolization before resection, and six patients also received concomitant intraoperative radiofrequency ablation of liver metastases.

**TABLE 1:**
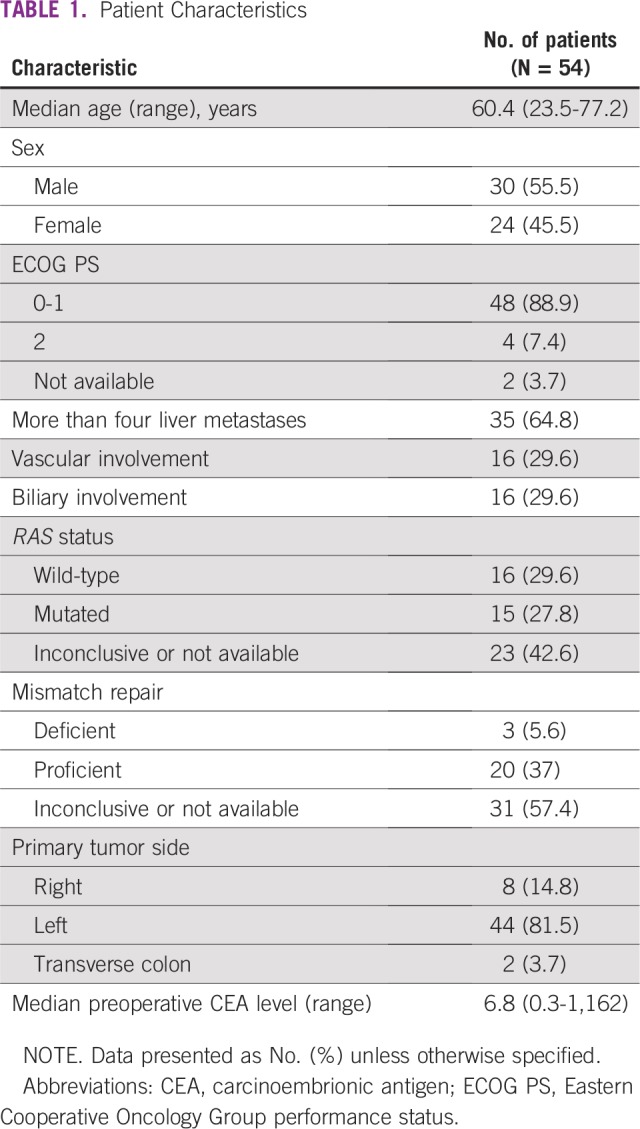
Patient Characteristics

### Efficacy and Safety

After metastasectomy, R0 resection was possible in 42 patients (77.7%). Moreover, conversion chemotherapy led to a complete pathologic response in six patients (11.1%), in whom no residual tumor remained in the pathologic analysis of the surgical specimen. Five patients (9.2%) had positive margins after surgery (R1 resection), and there was macroscopic residual disease (R2 resection) in seven patients (12.9%).

**FIG 1:**
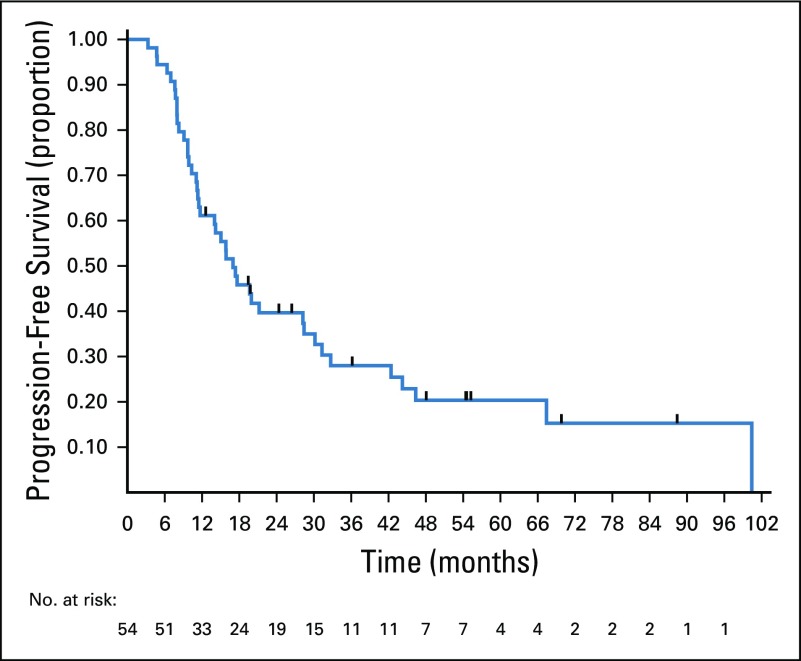
Progression-free survival of patients with borderline or unresectable liver metastases from colorectal cancer after treatment with conversion chemotherapy and surgical resection.

**FIG 2:**
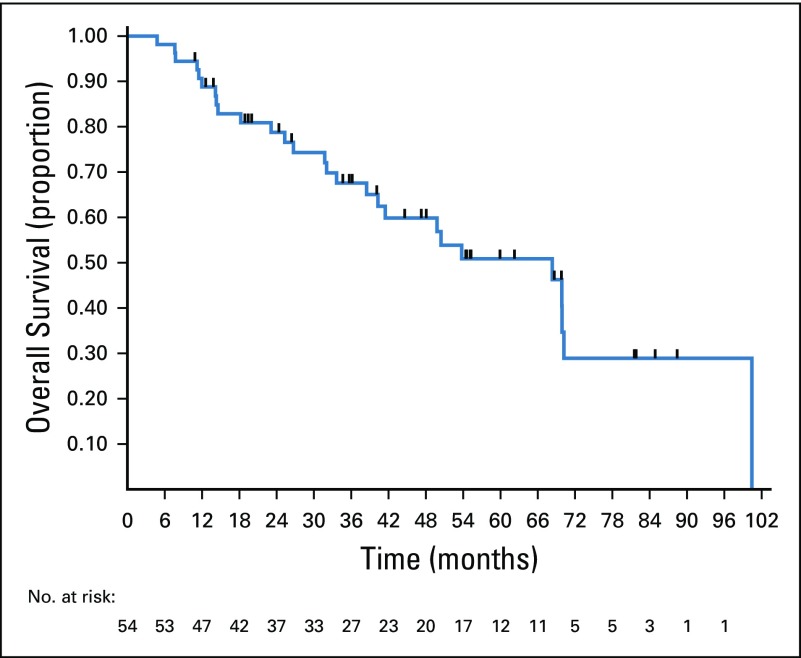
Overall survival of patients with borderline or unresectable liver metastases from colorectal cancer after treatment with conversion chemotherapy and surgical resection.

After a median follow-up of 37.2 months, 42 patients had disease progression or they died. Among these patients, seven died without recurrence. Median PFS was 16.9 months, and 3-year PFS rate was 28% (95% CI, 16.2% to 41%). Twenty-seven deaths occurred during the study period, with a median OS of 68.3 months. Three-year OS rate was 67.5% (95 CI%, 52.4% to 78.8%). The Kaplan-Meier curves for PFS are shown in Figure 1 and those for OS are shown in Figure 2.

Six patients remained alive with no evidence of disease after completion of at least 3 years of follow-up, and two patients remained alive after 5 years of follow-up. The only variable associated with PFS and OS was the result of the surgical resection. R1-R2 resections were associated with worse outcomes compared with R0 resection (PFS: hazard ratio, 2.65; 95% CI, 1.30 to 5.40; OS: hazard ratio, 2.90; 95% CI, 1.28 to 6.55). The results of the Cox proportional hazards model for prognostic factors are summarized in Table 2.

**TABLE 2:**
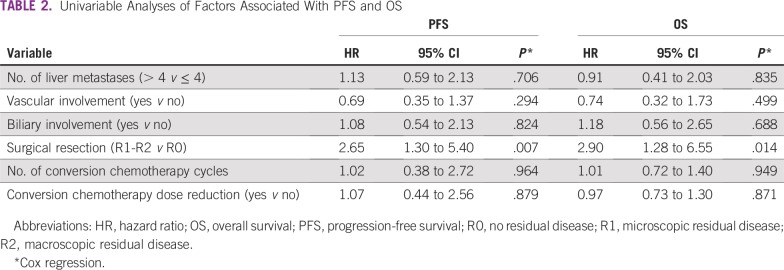
Univariable Analyses of Factors Associated With PFS and OS

Eight patients (14.8%) had complications after surgery potentially related to the conversion chemotherapy. Among these patients, three had liver failure, one of whom died as a result of liver failure. The other adverse events were infectious complications (two patients), upper GI variceal bleeding (one patient), acute pancreatitis (one patient), and acute myocardial infarction (one patient).

## DISCUSSION

Our results showed that conversion chemotherapy with mFLOX followed by surgical resection for B/U liver metastases from CRC was associated with a high rate of R0 resection (77.7%) and pathologic complete response (11.1%). Overall, this treatment strategy was safe, and patients had a favorable survival outcome with a median OS of 68.3 months. Another important finding of our study is that R0 resection is associated with better outcomes in terms of PFS and OS. This result highlights the importance of pursuing a complete resection in the patients that meet the resectability criteria.

Previous literature on conversion chemotherapy consists of retrospective studies, small phase II trials, and subgroup analyses of phase III trials. A phase II trial evaluating 44 patients with unresectable liver-only metastases showed that the FOLFOX regimen was associated with a 33% rate of R0 resection and a median OS of 26 months.^19^ Importantly, the results from that study (and from the others cited later in this section) refer to all patients who received conversion chemotherapy, including those who did not meet resectability criteria and did not receive surgery, which differs from the conditions in this study.

When irinotecan was added to fluorouracil, leucovorin, and oxaliplatin in the FOLFOXIRI regimen, the R0 resection after conversion chemotherapy ranged from 19% to 36% in prospective trials.^6,20,21^ In a subgroup analysis of a phase III trial that compared FOLFOXIRI and FOLFOX as first-line treatments for metastatic CRC, 36% of the patients with liver-only metastases treated with FOLFOXIRI underwent R0 resection compared with 12% of those treated with FOLFOX (*P* = .017).^6^ Similarly, in a phase II trial that evaluated bevacizumab plus FOLFOX or bevacizumab plus FOLFOXIRI for 82 patients with initially unresectable liver metastases from CRC, the R0 resection rates were 23% and 49%, respectively.^22^ However, that study did not clarify the role of the addition of bevacizumab in that scenario.

Regarding the use of EGFR monoclonal antibodies, phase II studies evaluated conversion chemotherapy with cetuximab or panitumumab combined with FOLFOX or FOLFIRI (infusional fluorouracil, leucovorin, and irinotecan) regimens.^23-26^ Results showed R0 resection rates ranging from 25.7% to 38% and median OS ranging from 29 to 49 months. One of the studies randomly assigned 138 patients with *KRAS* wild-type synchronous unresectable liver metastases to cetuximab plus chemotherapy (FOLFOX or FOLFIRI) or chemotherapy alone.^26^ The arm that received cetuximab had better R0 resection rates (25.7% *v* 7.4%; *P* < .01) and OS (median OS, 30.9 *v* 21.0 months; *P* = .013) than the chemotherapy alone arm. Finally, a small phase II trial evaluated 43 patients with unresectable liver metastases (69% of whom had confirmed *KRAS* wild-type tumors) and showed that conversion chemotherapy with cetuximab plus chrono-modulated irinotecan, fluorouracil, leucovorin, and oxaliplatin (chrono-IFLO) achieved an R0 resection rate of 60%.^27^

It is important to highlight that recent studies suggested that the laterality of the primary tumor influences the response to EGFR monoclonal antibodies; patients with right-sided tumors do not benefit from this treatment.^28,29^ Thus, it is possible that the laterality might influence decisions on the choice of conversion therapy. When used as a first-line regimen for metastatic CRC, the FOLFOXIRI regimen and the addition of bevacizumab or EGFR monoclonal antibodies to chemotherapy are associated with higher response rates.^8-12^ Together, this evidence and the results of the trials discussed here, suggests that these treatment strategies could be considered options for conversion chemotherapy. As mentioned previously, the response rate after conversion chemotherapy for unresectable liver metastases correlates with the resection rate.^13^

This study has limitations because of its retrospective character and the small sample size. Another important limitation is that we were not able to evaluate patients who received chemotherapy with the intention of conversion to surgery but who could not meet the resectability criteria. It is not possible to eliminate a selection bias in which patients with better response to treatment and more favorable prognosis were selected.

The strengths of our study are that, to the best of our knowledge, it is the first to report results of conversion chemotherapy with mFLOX, and we provide real-world data from public health care institutions in a developing country. Moreover, the approaches proposed are an example of how patient-centered treatment is possible and may lead to favorable outcomes even when resources are limited.

Of note, in a previous study from our institution, the use of mFLOX instead of FOLFOX as first-line therapy led to a cost decrease of R$13,000 (US$3,218) per patient for a treatment duration of 20 weeks.^17^ Addition of irinotecan (FOLFOXIRI) or monoclonal antibodies (cetuximab, panitumumab, or bevacizumab) increases treatment costs. In the future, the availability of monoclonal antibody biosimilars may allow treatment intensification at an affordable cost for limited-resources settings.^30,31^

In conclusion, treatment of B/U liver metastases from CRC with conversion chemotherapy using mFLOX followed by surgical resection was associated with favorable outcomes in terms of surgical outcomes and survival. In view of these results, mFLOX can be considered a low-cost option for therapy. Moreover, when resectability criteria are met, complete surgical resection should be pursued because it is associated with improved PFS and OS. Additional randomized studies will be required to confirm our findings.
